# The Nordic perspective on migration and empowerment

**DOI:** 10.1093/heapro/daaa021

**Published:** 2020-04-08

**Authors:** Berit Misund Dahl, Sofie Buch Mejsner, Leena Eklund Karlsson, Catrine Kostenius, Glenn Laverack, Heidi Myglegård Andersen, Maria Warne, Johan Lidmark

**Affiliations:** 1 Department of Health Sciences in Ålesund, NTNU-Norwegian University of Science and Technology, Âlesund, NO 6025, Norway; 2 Unit for health Promotion Research, University of Southern Denmark, Esbjerg, DK 6700, Denmark; 3 Department of Health Sciences, Luleå University of Technology, Luleå Campus, Luleå SE 97187, Sweden; 4 Visiting Professor, Department of Sociology and Social Research, University of Trento, Italy; 5 University College Absalon, Roskilde, DK-4000, Denmark; 6 Department of Health Sciences, Mid Sweden University, Östersund, SE-83125, Sweden; 7 County Council of Scania, Kristianstad, SE-29189, Sweden

**Keywords:** empowerment, health, migrants, Nordic, policy

## Abstract

International migration is a complex phenomenon that touches on a multiplicity of economic, social and security aspects affecting our daily lives. In the Nordic countries’ migration is a contentious political topic as the number of migrants has significantly increased in recent decades. The aim of this study is to analyse governmental policy documents on migrants in Denmark, Finland, Norway and Sweden and to identify and compare how they are described within an empowerment perspective. A critical discourse analysis was undertaken of each Nordic country. The findings revealed that all four documents placed migrants in a passive position in regard to decision-making and that an empowerment perspective was lacking. Migrants are similarly treated in each Nordic country as a problem to deal with rather than as a possible resource for the society and the approach seeks to protect the welfare state and the culture of the country. The lack of empowerment perspective may be having a negative impact on the health and well-being of migrants and on their integration in the Nordic society. The article concludes by raising several questions in regard to migration and empowerment in the Nordic context.

## INTRODUCTION

According to the World Migrant Report 2018 (McAuliffe and Ruhs, 2017) international migration is a complex phenomenon that touches on a multiplicity of economic, social and security aspects affecting our daily lives in an increasingly interconnected world. Migrants are considered at higher risk for health problems because of their irregular status and the consequences of economic and social marginalization (De Vito *et al.*, 2015).

The Nordic countries (Denmark, Norway, Sweden and Finland) have many similarities, for example, they share welfare states, with active labour market policies, and a universal approach to the delivery and financing of benefits and services (Greve, 2016). Migration and integration are currently highly contentious topics in political, public and scientific arenas as the number of international migrants have increased by 60% since 1990 (Pyrhönen et al., 2017). However, the number of migrants across the Nordic countries differs as a proportion of the population, for example, in Sweden it is 17.6%, in Denmark 11.5%, in Norway 15.1% and in Finland 6.2%. Comparatively, the average in Europe is 10.5% (International Organization for Migration, 2018). The three largest groups of migrants in Denmark and Norway come from other European countries, whilst in Sweden and Finland, it is Syrian and Iraqi migrants that are amongst the largest groups. The distribution of different reasons to receive permits to stay, as family reasons, work, study or asylum in the year 2018 has been put in a table (Table 1).

**Table 1:** Number of residence permissions in the Nordic countries and reasons for coming

2018^a^	Total number of positive decisions	Family reasons %	Work %	Study %	Asylum %	Other %
Denmark	76 156	12.0	45.0	33.0	2.0	7.0
Finland	25 538	37.4	32.7	17.8	10.6	1.5
Norway	36 915	34.4	40.5	11.4	12.6	1.1
Sweden	132 696	33.8	31.0	10.6	18.9	5.7

Reference: https://www.statistikbanken.dk/statbank5a/selectvarval/saveselections.asp; https://tilastot.migri.fi/index.html#applications/21205/59? l=en; https://www.ssb.no/innvgrunn/; https://www.migrationsverket.se/Om-Migrationsverket/Statistik/Beviljade-uppehallstillstand-oversikter.html (accessed 26 August 2019).

a The Finnish data are from July 2018 to July 2019.

The country that stands out the most is Denmark with much fewer residence permissions for family reasons and for asylum. It is also noteworthy that Sweden, having the highest total number of permits to stay, also has the highest share for asylum.

For the purposes of this article, the International Organization for Migration (IOM) definition of a migrant will be used as: ‘any person who is moving or has moved across an international border or within a State away from his/her habitual place of residence, regardless of (1) the person’s legal status; (2) whether the movement is voluntary or involuntary; (3) what the causes for the movement are; or (4) what the length of the stay is’.

## EMPOWERMENT: THE MEANS TO ATTAINING POWER

Empowerment is the means to attaining power. In the broadest sense, it is ‘the process by which disadvantaged people work together to increase control over events that determine their lives’ (Werner, 1988, p. 1). Most definitions give empowerment a similarly positive value and embody the notion that it must come from within an individual, group or community. Empowerment in this context therefore refers to policy that supports migrants at an individual and collective levels, in families and interest groups to facilitate empowerment, for example, through capacity building and skills development. Health promotion is often targeted at the individual and the risk is that this approach becomes superimposed in cross-cultural contexts where the family or community are considered to be a more important source of decision-making than the individual, including in the context of migrants. Community empowerment is an interaction between the individual, family and organizational forms of empowerment and is most consistently viewed as a continuum in which people become increasingly better organized and competent towards achieving social and political changes (Laverack, 2004).

Health policy is designed to deliver programmes that are professionally driven, top-down, pre-packaged and often with a focus on the bio-medical approach. Top-down is interpreted here as health needs that are decided by the top structures, usually a health agency, and are delivered ‘down’ to the intended beneficiaries. It is the health agency that holds the power through a control of the programme implementation, management and evaluation. The top-down approach has used epidemiological data to address a population-based agenda and this ‘lifestyle agenda’ has conveniently shifted the focus away from awkward political decisions that underlie poor health rooted in poverty and inequality (Labonte and Laverack, 2008). In contrast, bottom-up approaches assist the community to identify its own needs and to communicate these ‘up’ to the top structures of planning and decision-making.

The empowerment perspective in the context of this article involves the government, often through health promoting agencies, enabling migrants and asylum seekers to identify solutions to their own needs. For example, providing technical support to undertake a needs assessment to map migrant assets and resources so that they can be included in any ongoing programs. Other examples are building the capacity of community organizations working with migrants through skills training to better communicate and engage with individuals and groups and to provide basic resources such as communal spaces to allow migrants to meet and to discuss their concerns.

## AIM

The aim of this study is to analyse governmental policy documents on migrants in Denmark, Finland, Norway and Sweden and to identify and compare how they are described within an empowerment perspective.

## METHODOLOGY

Critical discourse analysis was used and views concrete social events and abstract social structures as part of reality (Fairclough, 2010). It distinguishes three dimensions between which there is a logical discourse: social events, social practices and social structures. The social events dimension is where the actual actions take place. The social practice domain controls which events are performed and social structures create the potential for action. There is a dialectic between the three dimensions (Dahl, 2017).

The inclusion criteria for selection of policy documents were one document from each Nordic country. Further the document was to be a key national policy document about migrants for each country, during the period 2017–2018. Four key governmental documents (Table 2) were undergoing analysis.

**Table 2:** Key Nordic policy documents on migration

Denmark	Finland	Norway	Sweden
The government: 1/3—2018: One Denmark without parallel society—No ghettos in 2030 (Regeringen, 2015)	Ministry of the interior: Government resolution on the future of migration 2020 strategy (Ministry of the Interior, Finland, 2013)	Ministry of justice and public security: White paper. From reception centre to the labour market-an effective integration policy (Norwegian Ministry of Justice and Public security, 2016)	Regeringens proposition 2016/17:1. The government: Budgetpropositionen för 2017, utgiftsområde 8 Migration (Sveriges Riksdag, 2016)

### Data analysis 

The methodology included a linguistic analysis of the critical discourse and a description of the social events of the texts (Fairclough, 2010). One author from each of the four countries reviewed a policy document and this was then shared with the other three reviewers to compare and contrast the findings. The following questions were put to the text: How are the migrants described in an empowerment perspective in the document and what words are used? A focus was also placed on interpretation of the relation between actual events and institutional practices. The researchers asked: Is the document linked to other documents? How are the sentences connected? The purpose was to reveal possible patterns about how migrants were described in the policy document. The third dimension of analysis was to explain the general social structures that had hegemony in the four documents. Questions to the text were: Can we reveal an empowerment ideology basis in the document? Is the document linked to a wider socio-political context of equity and does it focus on enabling the migrants to take control over the determinants of their health and well-being? The purpose was to unveil the hidden agendas and possible power dimensions in the policy documents.

## FINDINGS

### Findings from the Danish policy document

The Danish plan for reducing parallel societies emphasizes the importance of an ethos of a coherent Denmark with democratic values and where everyone actively participates. The document presents ambitions for improving the integration of newly arrived migrants and already resident non-western migrants in Danish society. The migrants are not described in an empowerment perspective or as being capable of contributing towards a plan for their integration into society. The responsibility for better integration into society is placed on the individual migrant although the municipality can also ‘play an important role in the conclusion of the parallel society’ (p.19). The text in the document identifies a top–down relationship with migrants and there is no description about migrant value or worth. The Danish approach uses an argumentative structure, with precise language in which the choice of words are forced actions such as ‘Parents are obliged to let their children participate in language teaching (…) The municipality is obliged to stop child support’ (p. 35). Modal auxiliary verbs such as ‘shall’ and ‘must’ are used to emphasize the statements towards the integration of migrants such as ‘Citizens in parallel society must be made to citizens who contribute to society’ (p. 7) and ‘you shall not be able to move into a ghetto area’ (p. 7). For cohesion between sentences and to construct a greater unity towards the government’s’ ideology, pronouns are used for example when ‘have for decades accepted too many refugees and as a society, we have not set the necessary demands’ (p. 5). These examples illustrate the top-down approach that provides only a passive role for migrants even though it recognizes that ‘far more people are ready to take a job’ (p. 6) and that the government insists that ‘citizens seize opportunities and take on the values and norms’ (p. 9) of the Danish society. A way forward using an inclusive dialogue and a participatory approach with migrants is, however, not discussed.

### Findings from the Finnish policy document

The Finnish document uses concepts of migration and mobility in general terms and draws on arguments for the enhancement of the sustainable economic growth of Finland, where ‘migration will enhance the wellbeing of the population and boost Finland’s competitiveness’ although migration ‘will be kept foreseeable and controlled’ (p. 4). The document highlights the need of attracting young migrants to Finland as it has a positive impact on the age structure of the population and that young adults are expected to fill the gaps in the labour market: ‘Finland needs young migrants … who enter the labour market … to supplement the Finnish labour force’ … ’Short-term and longer term migration of skilled labour into Finland must be promoted, particularly by developing forecasts of labour needs and the resources for targeted recruitment abroad’ (p. 13). In parallel with the discourse of migrants as a tool for ensuring international competitiveness, societal development and economic growth of Finland, there is a discourse on an image of being an open and safe country that fulfils its obligation to safeguard migrants that need special protection: ‘Under international agreements, the human rights of persons unlawfully present in Finland or present in Finland without a residence permit must be guaranteed’ (p. 15).

The document stresses the responsibility of the migration authorities to be effective and transparent in handling the matters of migrants and to ensure that the specific needs of vulnerable people are met. The document calls for clarity of the division of tasks and responsibilities and the cooperation with various local and central entities dealing with migrant issues. Migrants are articulated to be in need of guidance from the authorities although ‘opportunities for migrants to participate in society must be supported through special measures, and information on must be provided more actively and through more channels’ (p. 18). However, the strategies for these special measures are lacking in the document and it does not provide suggestions on how to overcome the dominance of negative views of migration among the Finnish population, who see ‘the migration as a threat to national culture’ (p. 9).

The document draws on legislation, acts, statistics, selected research reports and international regulations to support an argumentative dominant discourse. The text focuses on the necessity of ‘mobility’ to Finland but also strengthens the paternalistic ideology of protecting and integrating migrants due to international agreements and national obligations. The text indicates expert power-over to migrant issues even though it recognizes that ‘the views of migrants themselves must be sought’ (p. 22). The document is lacking in suggestions for actions to facilitate the migrants to take control over the determinants of their health or enabling their voices to be heard. The Finnish document includes many subordinate clauses with the modal auxiliary verb ‘will’ such as ‘Daily dialogue between different population groups will help migrants find a role’ (p. 18) followed by explicit obligational modalities with imperative modal verb ‘must’, ‘…the ability of Finnish society to support the participation and integration of migrants must be strengthened’ (p. 18). With respect to transitivity, the text uses a linguistic feature of nominalization whereby nouns migration or *mobility* (a phenomenon) stands for the process instead of *migrant* (subject). These examples indicate that the Finnish policy document does not use an empowerment and is a top-down articulation of measures ensuring Finland’s competitiveness in which migrants must bring with them innovative ways of doing things.

### Findings from the Norwegian policy document

The Norwegian document uses arguments about the Norwegian welfare model that ‘is dependent on high participation in the workforce’ (p. 5). The intertextuality focuses on the welfare of Norwegians and on maintaining the Welfare state using an argumentative structure that presents ambitions for the integration of migrants. The focus is on how migrants can get a job or education immediately after arrival in Norway and the responsibility and agency is given to them to collaborate with institutional entities. Although the cooperation between the government and the regional councils and municipalities provides a structure for support, the perceived role of the migrant is positioned in a passive role. The use of modally auxiliary verbs ‘shall’ and ‘must’ in the text strengthens this position: ‘Everyone shall provide for themselves’ … ‘Immigrants who come to Norway must adapt to a totally new set of circumstances’ (p. 11) The migrants are not described within an empowerment perspective and it is a top-down approach that dominates the discourse: ‘immigrants and their children (must) contribute to and participate in their communities’ (p. 17). Supportive strategies related to establishing a dialogue with migrants and developing coping resources are absent in the document. The dominating discourse is about upholding the welfare state, for example, (migrants) ‘provide the municipality with both the necessary workforce and tax revenue’ (p. 14) and that the Norwegian experts know what is best for migrants. The individual migrant is only seen as a worker and a taxpayer, not as an individual with personal challenges, rights or specific needs.

### Findings from the Swedish policy document

The Swedish document recognizes ‘foreigner’s’ rights to travel in and reside in Sweden and for the reception of asylum seekers. In the Swedish document, the focus is on refugees and migrants in general are viewed in terms of their coming in or leaving the country generating a flow of things or people that cost money labelled as ‘unit-costs’. There are no descriptions about the value or worth of migrants or their potential to contribute to Swedish society, for example, through work or paying taxes. The migrants are described passively, in need of protection underpinned by the importance of international agreements: ‘A strengthened and constructive cooperation within the EU as well as globally is a cornerstone in a long term sustainable migrant policy’ (p. 28). The situation in Sweden is dependent on how other countries act and that some do not take appropriate responsibility for the movement of migrants in Europe. Referring to international agreements makes it easier for the Swedish government to switch from one of Europe’s most generous migrant policies to the EU minimum level and still argue in favour of a generous migrant policy: ‘Sweden will in the future still be one of the EU countries taking on a larger responsibility as for migrant reception’ (p.28).

The Swedish document gives migrants a passive position and objectifies them with no empowerment perspective, although it is made clear that they do have rights according to international agreements. It state that ‘Sweden should continue to be a strong and important advocate for asylum rights and vulnerable groups’ and that ‘In the current situation with distresses on the asylum system there is a risk of vulnerability particularly for women and girls’ (pp. 28–30). The document advocates for a top-down perspective, for efficiency, cost reduction and that the government is in control. These are its dominant features with a concession to the necessity of some repressive actions in order to minimize the number of migrants coming to Sweden and to make those that already have come, to leave. The document advocates that the present migrant policy is generous and humane and: ‘The Government’s assessment is that the Migration Board’s efforts, given the exceptional circumstances prevailing, contributed to a well-balanced operation’ (p. 26).

## DISCUSSION

### Cost and contribution to society

The four policy documents present a review of how migration has been managed in each Nordic country to seek to protect the welfare state model (Table 3). The vision of the Nordic welfare state has traditionally been to provide generous benefits in a universalistic perspective. However, migration threatens to destabilize the welfare model and the policy documents present a similar top-down, political position on how to avert public fears and to find a ‘solution’ to the migrant issue.

**Table 3:** Summary of the document discourse on migration and empowerment

	Migrant empowered or passive?	Dominating discourse	Empowerment perspective
Denmark	Passive position	Top-down	Lacking
Finland	Passive position	Marketization/business discourse	Lacking
Norway	Passive position	Top-down	Lacking
Sweden	Passive position	Generous refugee policy	Lacking

However, the documents also offer some aspects of hope for future migration in the Nordic countries. In Denmark, the government has published a new policy which is a strict and consistent immigration policy to ensure an open Denmark for those ‘who can and will’ and to shut the doors for those ‘who do not want to’. The government’s first step is to immediately make proposals to make it less attractive to apply for asylum in Denmark and at the same time the government will present a plan to improve efforts in the ‘ghetto areas’ (Regeringen, 2015). In Finland, the overall political aim is to pave the way for a more active migration policy and to ensure that migration issues are given thorough consideration in Finland’s public policy. The future focus is on increasing the employment rate among migrants residing in Finland. (Ministry of the Interior, Finland, 2013). In Norway, the political aim is for more migrants with refugee background to find jobs faster and to stay employed. The Norwegian welfare model is dependent on high participation in the workforce and a reduction in the active workforce can challenge the system in terms of its sustainability and legitimacy (Norwegian Ministry of Justice and Public security, 2016). The Swedish approach aims to ensure a long-term sustainable migration policy that protects the right of asylum seekers and has regulated immigration that facilitates mobility across borders, promotes one demand-driven labour migration and takes into account the developmental effects of migration, as well as deepening European and international cooperation (Justitiedepartementet, 2018).

Overall the four governmental documents address migrants in general terms. In the Finnish and the Norwegian documents, the labour market is especially in focus but since the migrants are given a passive role in the documents potential differences between different categories of migrants are not addressed, although the focus on the labour market is an example where it very well could have been done.

### Migrants as objects

The analysis of the four documents has revealed that the expert discourse is predominant and that migrants are construed as passive receivers of services having very limited influence and with a lack of a clear political vision in regard to integration (Wimelius et al., 2017). Migrants are often objectified and treated as economic commodities, for example, through paying taxes. There are few descriptions about the value or worth of migrants and how they can contribute in a positive way to the social fabric of Nordic society. Instead, the migrants are described in terms of the need to protect them, although it is not necessarily clear from what they need to be protected. Migrants are viewed as an asset in the Finnish, Norwegian and Danish documents. Finland seeks opportunities through the labour market and contributions to the welfare state (Greve, 2016). However, there is a fundamental difference between migrants as a cost in Sweden and as a contribution to the economy in Finland, Norway and Denmark. Migration is definitely seen as a threat to the Nordic culture and inclusion into society is only acceptable if the migrants can positively contribute with their labour and become tax payers. If not, migrants are viewed as being a threat to the welfare system, which has to be protected.

Overall, the documents present a top-down approach. In Finland, this is presented a little differently as a marketization and business discourse and in Sweden as a seemingly generous refugee policy. However, an overall empowerment ideology was found to be lacking in all four Nordic policy documents.

### Limitations

Greenland was not included in the study because it is a protectorate of Denmark and covered by the same policy document. Iceland was not included in the study because no policy document was found by the authors. It was not possible to find identical documents for each Nordic country although it was found to be possible for the authors to identify similar documents that could be compared to reveal the dominant discourses and political positions that are presented in each country. The data consists of one policy document on immigration for each country. To find out whether the countries have policies and practices that promote empowerment, the countries’ practices should also be studied; however, this was not the aim of this study. The documents were written in official language of each country; however, three of the documents were translated into English (the Danish, Finish and Norwegian). Thus, language and semantics can be seen only as a small limitation of the study.

## CONCLUSION

This study has illustrated that migrants are not described in an empowerment perspective in four key political documents in the Nordic context. Very little agency is given to migrants and they are treated as a problem to deal with rather than a possible resource for the society. The governmental policy documents on migrants do not support social inclusion and integration. The lack of an empowerment perspective could be having a negative impact on the well-being of migrants and could be inhibiting their integration into Nordic society. The way in which the migrants are constructed in the policy documents could also be having consequences for the individual and collective levels of control and consequently for health outcomes.

Although there are subtle differences in the four countries the study points to general similarities in approach within the Nordic context in that they all seek to protect the welfare state and the culture of their country. The integration of migrants into society could be perceived as a threat and therefore they are given a passive position in decision-making and are not being empowered to take more control of their lives, and health.

The study raises several key questions that need to be addressed in the future:

Is government policy in the Nordic countries purposefully excluding migrants despite a seemingly generous package of benefits?

Is government policy in the Nordic countries putting the welfare model before the rights and needs of migrants?

Is government policy in the Nordic countries putting its cultural values before the rights and needs of migrants?

Is government policy in the Nordic countries having a negative impact on the health and wellbeing of migrants?

How does the present policy discourse influence the practice level in regard to the empowerment of migrants?
